# The 'Ziran' wrap: reconstruction of critical-sized long bone defects using a fascial autograft and reamer-irrigator-aspirator autograft

**DOI:** 10.1186/s13037-014-0040-7

**Published:** 2014-10-04

**Authors:** Navid M Ziran, Wade R Smith

**Affiliations:** Hip & Pelvis Institute, 2001 Santa Monica Blvd, Suite 760, 90404 Santa Monica, California USA; Mountain Orthopaedic Trauma Surgeons at Swedish, 701 E. Hampden Ave, CO 80113 Englewood, UK

**Keywords:** Critical-sized defect, Stem/progenitor cells, Guided bone regeneration, Periosteum, Reamer-irrigator-aspirator

## Abstract

Reconstruction of critical-size bony defects remains a challenge to surgeons despite recent technological advances. Current treatments include distraction osteogenesis, cancellous autograft, induced membranes (Masquelet procedure), polymeric membranes, and titanium-mesh cages filled with bone graft. In this article, the authors presents two cases in which critical-sized defects were reconstructed using a meshed fascial autograft encasing reamer-irrigator-aspirator (RIA) autograft and cancellous allograft. This article will discuss the clinical outcomes of the technique, comparison to other current techniques, and technical insight into the potential biological mechanism.

## Background

Critical-sized bony defects are defects of bone that do not heal on their own. There are numerous methods utilized to regenerate these defects including: use of polymeric membranes, the Masquelet procedure, titanium cages, allo/autograft, distraction osteogenesis, and free microvascular fibula transplant. This article demonstrates a technique utilized in two patients to treat large bony defects. Both patients had a satisfactory clinical outcome. The authors recognize this method is not standard of care but demonstrate this technique to encourage further discussion in managing these difficult injuries.

## Case one

**Figure 1:**
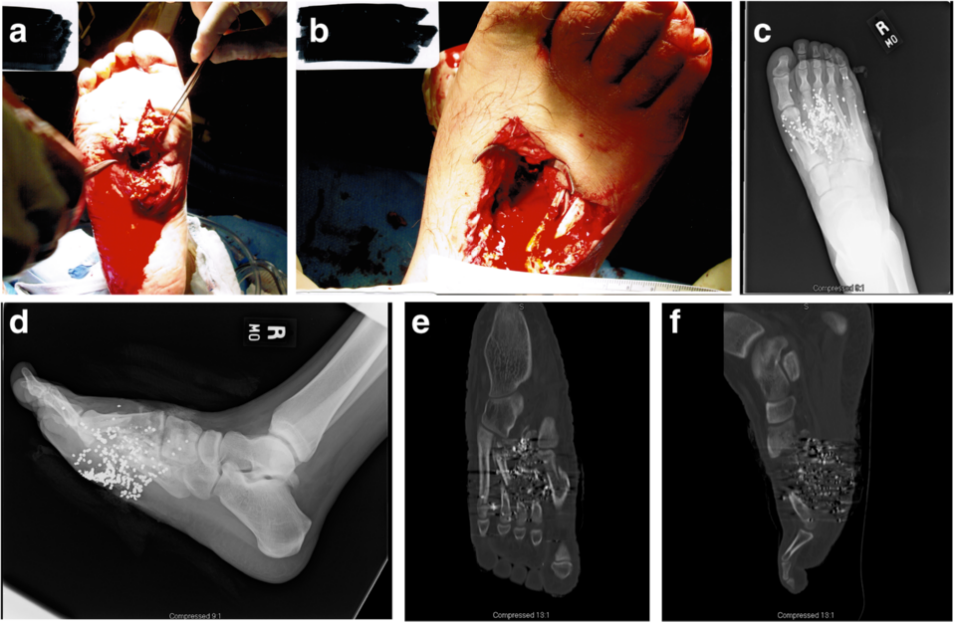
**Photographs (a-b), radiographs (c-d), and CT (e-f) of the through-and-through shotgun injury to the right foot.** Radiographs **(c-d)** and CT reconstructions **(e-f)** demonstrate significant buck-shot, first metatarsal fracture, comminuted 2^nd^ and 3^rd^ metatarsal fractures, fourth metatarsal fracture, fifth metatarsal neck fracture, and comminuted fractures of the intermediate and lateral cuneiforms (not shown).

The patient is an eighteen-year old male who sustained an isolated, self-inflicted through-and-through shotgun wound to his right foot (Figure 1a and b). He was evaluated in the emergency room (ER) where antibiotics and tetanus were administered and was taken urgently to the operating room (OR) for an irrigation and debridement (I&D). The patient had a significant amount of buck shot in the dorsal and plantar aspects of the right foot (see Figure 1c-f). The posterior tibial pulse was palpable but no pulse in the dorsalis pedis artery was present. Initially, the patient had no plantar or dorsal sensation from the midfoot distally. The following fractures were noted: comminuted fracture of the first and fourth metatarsals, fifth metatarsal neck fracture, comminuted fractures of the intermediate and lateral cuneiforms, and an approximate 4–5 cm bony defect of the second and third metatarsals with only the distal aspects of both metatarsals intact.

After the initial I&D, the plantar wound was primarily closed and a vacuum-assisted closure (VAC) was placed on the dorsal wound. Three subsequent I&Ds with VAC exchange were performed over the following 10 days. During the second I&D, the first metatarsal was closed reduced and percutaneously pinned since it was dorsally translated on the midfoot. The fourth and fifth metatarsals were pinned to the cuboid to prevent lateral translation of the forefoot since the Lisfranc ligament and base of the 2nd metatarsal were not intact. The definitive surgery was then performed to reconstruct the 2nd and 3rd metatarsals approximately three weeks after the initial injury.

**Figure 2:**
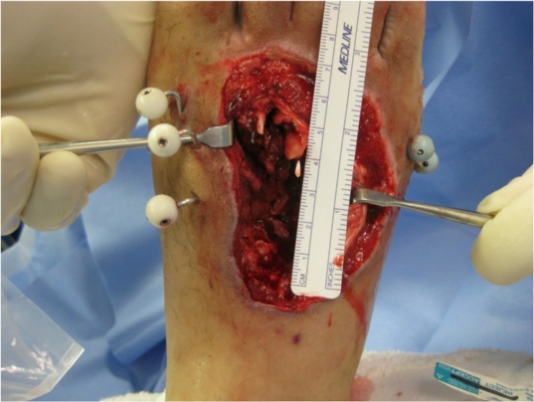
**Photograph of the right foot demonstrating the bony defects of the 2** ^**nd**^ **and 3** ^**rd**^ **metatarsals.**

**Figure 3:**
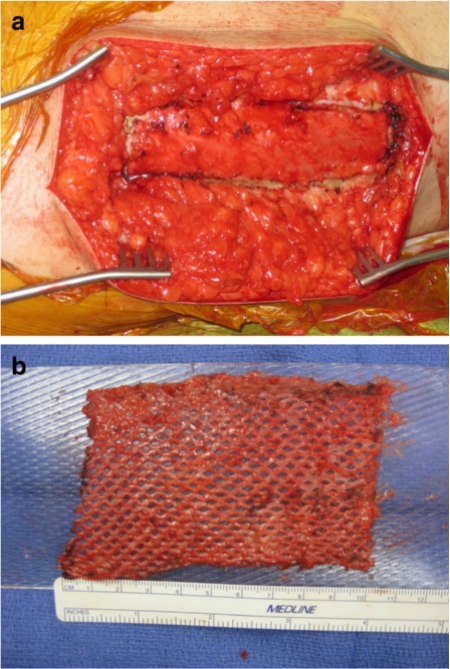
**Right iliotibial band donor site (a) and after fenestration (b).**

**Figure 4:**
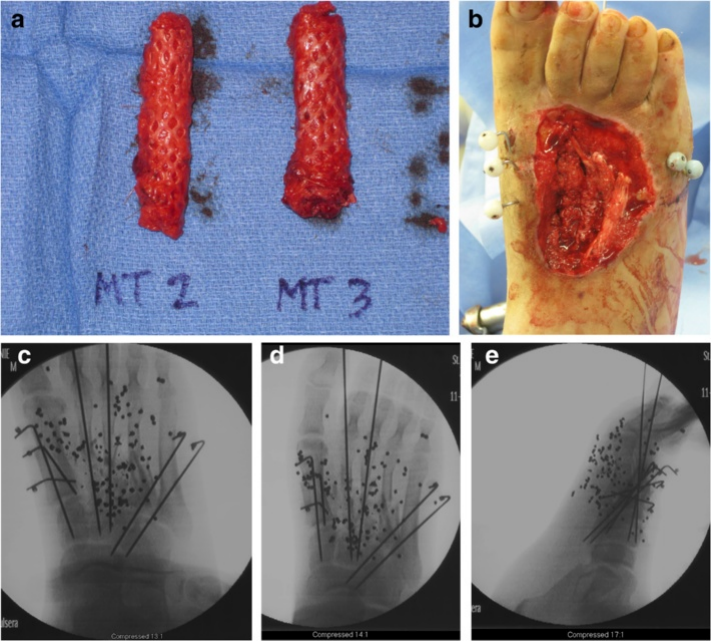
**Intra-operative photographs (a-b) and radiographs (c-e) of the right foot.** Final fascial graft encasing autograft, cancellous allograft, and BMP-2 (A) for each metatarsal. Figure 4b demonstrates *in situ* placement of the grafts, with corresponding intra-operative fluoroscopic anteroposterior (AP), oblique, and lateral views **(c-e)**.

Definitive reconstruction was performed in the following manner. First, the dorsal wound was debrided back to healthy, bleeding tissue. The extensor hallucis longus was intact. The extensor digitorum longus to the second and third toes were not intact. Two 1.6 mm Kirschner wires were first inserted retrograde through the distal portion of the 2nd and 3rd metatarsals (Figure 2). In order to reconstruct the missing bone segments of the second and third metatarsals, reamer-irrigator-aspirator (RIA) bone graft was obtained from the patient’s right femur in the following manner. A drill-tip guide wire was inserted percutaneously into the piriformis fossa, and the wire tip position was confirmed using intra-operative fluoroscopy. The outer cortex was then drilled, a guide wire was inserted down the intramedullary canal, and two reaming passes were performed using 12 and 13 mm reamers. A sufficient amount of bone graft was aspirated from these two passes. The incision was irrigated and closed with staples. After the RIA bone graft was obtained, the fascial graft was harvested. A direct lateral approach to the right thigh was performed down to the iliotibial band (ITB). Based on pre-operative planning measurements, approximately 6 cm length × 2.5 cm width of ITB was sharply excised (Figure 3a) in a rectangular shape. The ITB was closed with #1 Vicryl suture without tension. The skin was closed with 2–0 Vicryl and staples. The fascial graft was then placed on a skin graft mesh board with the interior (medial) fascial portion facing up; the graft was meshed using 1:1.5 board (Figure 3b). Finally, the tubular graft was prepared by placing RIA autograft, approximately 2–3 cc of cancellous allograft (Musculoskeletal Transplant Foundation), and 5 mg of BMP-2 (Medtronic, delivered as in a synthetic collagen vehicle) on the fascial graft to make a “wrap.” The fascial graft “wrap” was then closed into the shape of a cylinder and closed with 3–0 Monocryl suture (Figure 4a). Two such grafts were prepared for the second and third metatarsals.

Lastly, the cylindrical autografts were interposed in the bony defects of the 2nd and 3rd metatarsals, and the Kirschner wires were gently advanced retrograde. The K-wires both achieved purchase in the midfoot attempting to place the 2nd and 3rd metatarsals “wraps” in their anatomic position relative to the intermediate and lateral cuneiforms, respectively (Figure 4b-e). Anatomic placement of the reconstructed 2nd and 3rd metatarsals was difficult secondary to comminution of the cuneiforms. After the procedure, final intra-operative images were obtained. The dorsal wound was covered with Xeroform dressing and sterile sponges. The plantar wound, which had subsequently dehisced slightly from the original primary closure, was packed with moist Kerlix and underwent twice a day dressing changes after 48 hours.

Four days later, the patient underwent a latissimus free-flap coverage to the dorsal and plantar foot wounds along with skin grafting by plastic surgery. Post-operatively, the flap was monitored closely for 24 hours. The patient remained non-weight-bearing for 8 weeks. X-rays done at 3 weeks (not shown) demonstrate no change from intra-operative fluoroscopic images. The pins in the 1st, 4th, and 5th metatarsals were removed in clinic at approximately 6 weeks, and the 2nd and 3rd metatarsal pins were pulled at approximately 8 weeks. The patient was made weight bearing as tolerated in a walking boot at the 8 weeks post-operative visit.

**Figure 5:**
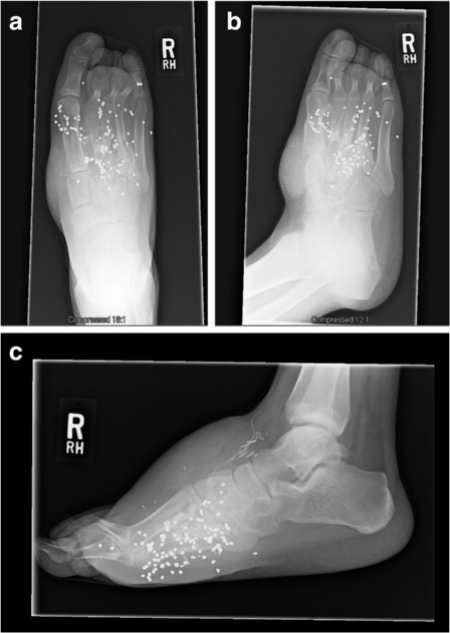
**AP (a), oblique (b), and lateral (c) radiographs of the right foot 4.5 months post-op.**

**Figure 6:**
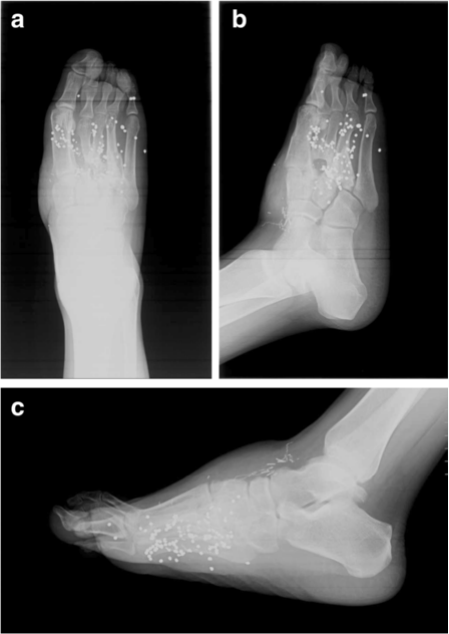
**AP (a), oblique (b), and lateral (c) radiographs of the right foot 7 months post-op.**

**Figure 7:**
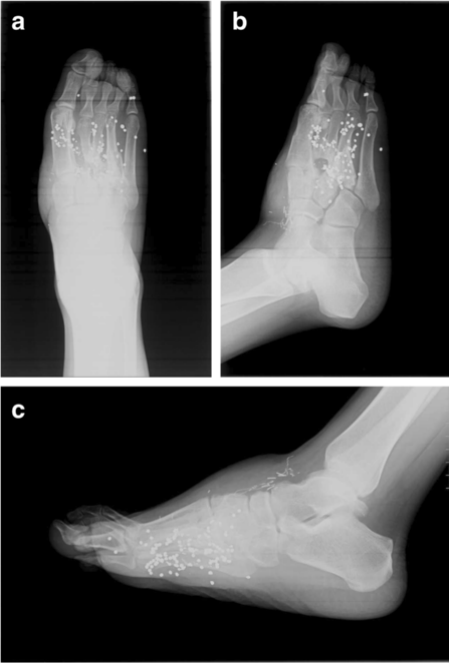
**AP (a), oblique (b), and lateral (c) radiographs of the right foot approximately 3 years post-op.**

**Figure 8:**
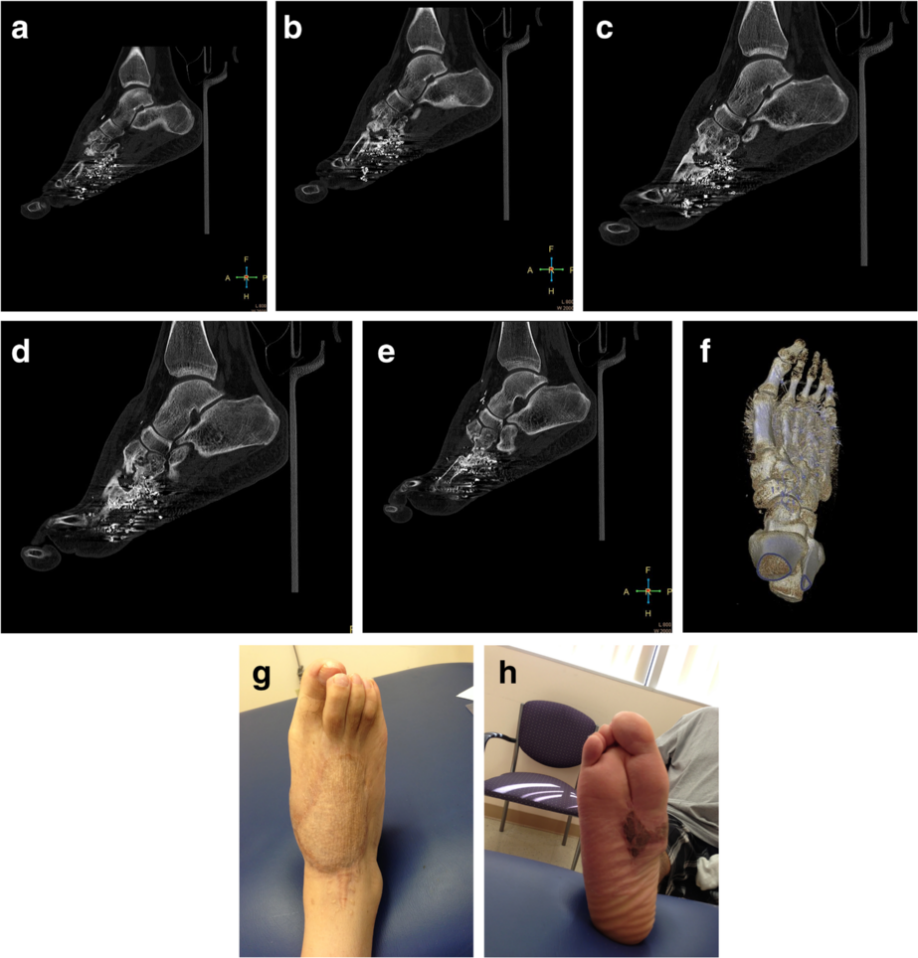
**Computed tomographic (CT) and photographs of the right foot. Sagittal (a-e) and CT reconstructions (f) of the right foot approximately 3 years post-op.** Photographs **(g-h)** of the right foot 3 years post-op.

The patient was subsequently incarcerated. He was seen at 4 ½ months post-op, but based on radiographs (Figures 5a-c), it was difficult to discern the quantity and shape of bony regeneration. At that time, he was ambulating with minimal pain. At 7 months post-op, he complained of intermittent pain with heavy activity and alleviated by rest. He mentioned that he was able to hike two miles in his cam walker boot. He had no antalgic gait, and was able to fit into a normal shoe. Radiographs at 7 months post-op demonstrate a second and third metatarsal abutting against the midfoot (Figure 6a-c). The patient was seen again approximately 3 years post-op, had a normal gait, and complained of mild pain after prolonged standing. He was able to wear normal shoes. Radiographs (Figures 7a-c) and CT (Figures 8a-f) demonstrate that the second and third metatarsal regenerates migrated proximally, fused together, and abutted against the midfoot. A gross photograph of the foot (Figure 8g-h) demonstrates proximal and dorsal migration of the second, third, and fourth lesser toes with subsequent narrowing of the foot. Despite this deformity, the patient had no skin breakdown or significant pain with ambulation.

## Case two

**Figure 9:**
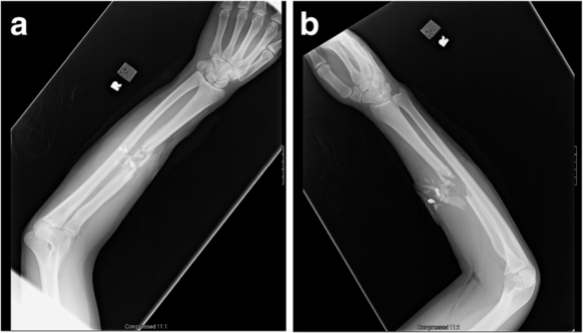
**AP (a) and lateral (b) radiographs of the right forearm.**

The patient is an 18-year-old male who sustained a close-range through-and-through gunshot wound to his right forearm from a .45 caliber handgun. He presented to the ER with an isolated, comminuted right radial shaft fracture (Figure 9a-b). Sensation was diminished to light touch in the median and ulnar nerve distribution but intact to light touch in the radial nerve distribution. He had 2+ radial and ulnar pulses. The patient underwent bedside I&D in the ER and received 24 hours of antibiotics.

The radial shaft defect was managed in one operative session using RIA autograft, cancellous allograft, and meshed fascial autograft. The patient was placed supine on a radiolucent table, and the right lower extremity was prepped and draped in sterile fashion. RIA autograft was obtained from the right femur as described for the previous case. A sufficient amount of bone graft from the right femur was obtained.

Next, a direct lateral approach was performed on the right femur through skin and subcutaneous tissue down to the ITB as previously described. Based on a prior measurement of the radial shaft defect, a rectangle of the iliotibial band was excised. The ITB excision was then meshed using a 1:1.5 mesh board, similar to preparation of a skin graft. The ITB was then closed without tension. The subcutaneous tissue was closed using #1 Vicryl and the skin was closed with 2–0 Vicryl and staples. A sterile dressing was applied.

**Figure 10:**
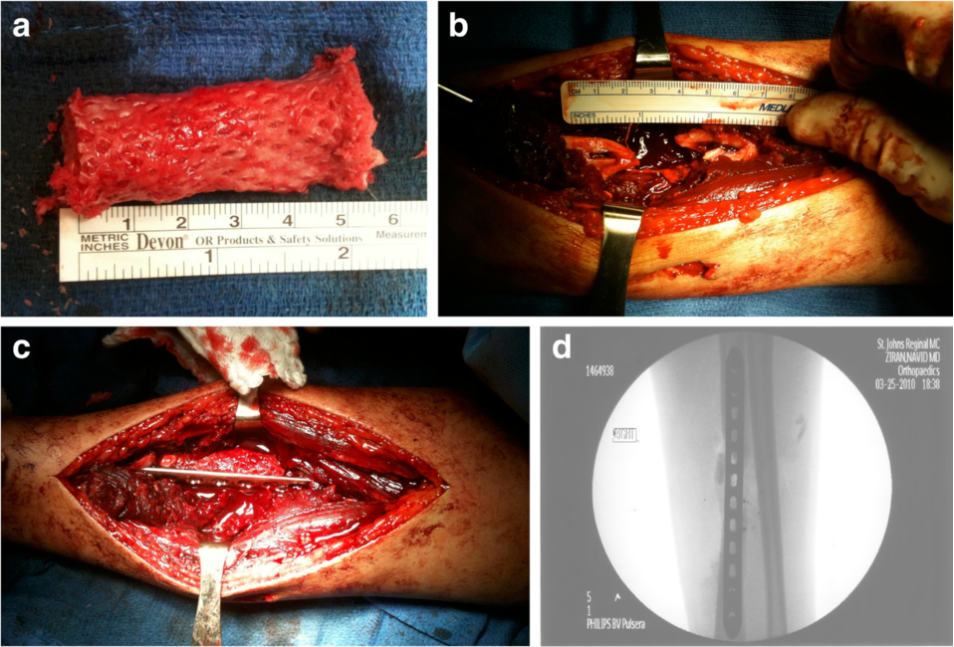
**Intra-operative photographs and radiograph intercalary fascial autograft of the right radial shaft.** Fascial autograft encasing cancellous autograft and allograft **(a)**. Intra-operative photo demonstrating the size of the right radius bony defect before **(b)** and after **(c)** intercalary graft placement and fixation. Intra-operative AP fluoroscopic view of the right forearm **(d)**.

After ITB harvest, fluoroscopic views of the left wrist were done to check the ulnar variance. The right upper extremity was then prepped and draped in sterile fashion. The ITB was then placed exterior side down, RIA bone graft and cancellous allograft placed on the fascia, and the fascia was rolled into the shape of a cylinder. This “wrap” was then closed with 3–0 Monocryl and sized to the defect (Figure 10a).

Lastly, the midshaft radius was exposed using the volar approach of Henry. There was significant damage to the flexor pollicis longus from the gunshot wound. After exposure of the radial shaft, RIA bone graft was then inserted into the ends of the intramedullary canal of the radius defect, and the intercalary “wrap” was placed in the defect. Care was taken to ensure overlap of the defect with the ends of the bone. The rotation of the radial shaft was provisionally aligned during placement of the intercalary graft by supinating the forearm and holding the two fracture ends with clamps. A 3.5mm LC-DCP plate was secured to the proximal portion of the fracture. The length of the radius was determined by fluoroscopic comparison of the ulnar variation to the contralateral side. Alignment was determined clinically and fluoroscopically, using the plate as a reduction tool. Rotation was determined by using the plate as a reduction tool (the plate rested on the flat volar aspect of both ends of the radial shaft). The plate was then secured to the distal fragment (Figure 10b-d). Four cortices of fixation were obtained proximal and distal to the fracture. A long plate with a large working length was used to ensure motion at the defect site, consistent with bridge plating technique. Pronation and supination were checked prior to closure to ensure that no significant malrotation deformity existed. The skin was then closed with 2–0 Vicryl and staples.

**Figure 11:**
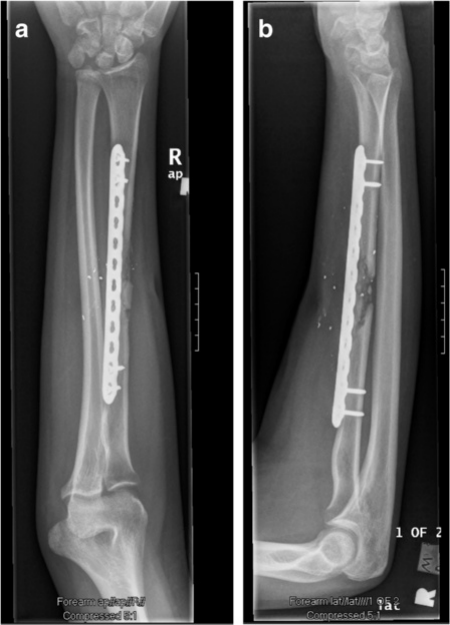
**AP (a) and lateral (b) radiographs of the right forearm 2 months post-op.**

**Figure 12:**
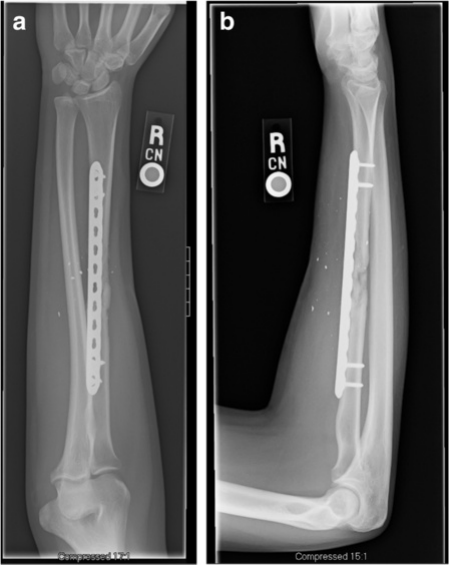
**AP (a) and lateral radiographs (b) of the right forearm 4 months post-op.**

**Figure 13:**
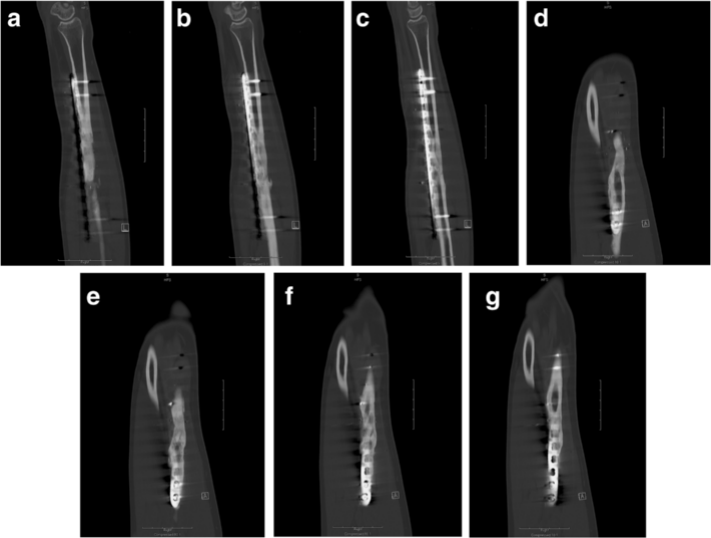
**Sagittal (a-c) and coronal CT (d-g) reconstructions of the right forearm one year post-op.**

The patient was placed in a posterior splint for 2 weeks at which point staples were removed from the thigh and forearm. The patient was unable to attend therapy due to lack of insurance and was thus instructed on gentle pain-free range of motion exercises, including pronation, supination, wrist/elbow flexion/extension, and digital range of motion. He was also instructed to begin gentle loading of the forearm over the ensuing 2 months. Radiographs were taken at 8 weeks demonstrating callus formation (Figure 11a-b). At 16 weeks, radiographs demonstrate further consolidation of the fracture (Figure 12a-b). Clinically, the patient had no pain and full pronation and supination at 16 weeks. Sagittal (Figures 13a-c) and coronal (Figures 13d-g) computed tomographic (CT) scan reconstructions at one year were performed for research purposes. They demonstrate bridging bone and cortical remodeling; the fenestrations of the fascial graft are also identified on the CT scan. The bone regenerated both endosteally and periosteally in relation to the fascial graft.

### Technical challenges – safe surgical technique

In the forefoot reconstruction, the 2nd and 3rd toes migrated dorsally and proximally; this issue is predominantly a cosmetic issue for the patient since the deformity does not cause significant pain, skin breakdown, or affect his ability to fit into shoes. The proximal migration of the reconstruction is due to the lack of proximal bony support against the midfoot as well as compromise of the dorsal/plantar metatarsal ligaments and deep transverse metatarsal ligaments. Thus, the reconstructed tubes “telescoped” on the K-wires and migrated proximally. This proximal and dorsal migration could have been prevented by fixation of the intact 2nd and 3rd metatarsal heads to the 1st, 4th, and 5th metatarsal heads (i.e., Kirschner wires or external fixation). Based on the CT scans done at 9 months (not shown) and 3 years post-op, it appears that the second and third metatarsal grafts regenerated by nine months, but the third metatarsal graft had “kinked,” potentially due to proximal migration. If the distal 2nd and 3rd metatarsals were maintained out to length with a Kirschner wire traversing the distal aspects of the 1st through 4th metatarsals), the second and third rays may not have migrated dorsally and proximally. Such a K-wire may be hard to place due to the concavity of the metatarsal arch; instead a 1st to 2nd metatarsal K-wire and a 5th-4th-3rd metatarsal K-wire could also have been utilized. Since the length was not maintained, the 2nd and 3rd metatarsals migrated proximally and dorsally, and the forefoot narrowed. The majority of the narrowing of the foot occurred after the patient began weight bearing likely as the 1st and 4/5th metatarsal compressed the 2nd and 3rd metatarsals. A dorsal external fixator may have been another option to maintain foot width. Nevertheless, despite the above-mentioned deformities, the patient had a plantigrade biologic extremity, minimal pain with weight bearing, and was satisfied with the treatment.

There were no significant challenges in the radial shaft reconstruction. The radial length was established, as mentioned before, by matching the ulnar variance of the opposite side. Alignment was evaluated fluoroscopically, and rotation was evaluated by placing the plate on the volar portion of the proximal and distal radial shaft; in addition, the forearm was pronated and supinated to ensure satisfactory rotation in each direction. The bony defect was bridge plated, and the patient was encouraged to apply a small compressive load to the radius two-weeks post-operative.

## Discussion

A critical-size defect is defined as the smallest bony defect that does not heal spontaneously and is generally defined as 6 cm. However, it is more logically defined in the context of the bone, with a critical-sized defect defined by multiplying the shaft diameter by 2.0 – 2.5 [1]. There are numerous treatment options for these defects based on the anatomic location. In this article, the authors describes a single-step technique, which entails placement of cancellous autograft, cancellous allograft, with or without BMP-2 in an autologous fascial fenestrated “wrap” to reconstruct critical-sized defects in the radius and metatarsals. The authors recognize that this technique, demonstrated in two patients, is not within standard of care. The purpose of demonstrating this technique is to present another potential option that can encourage discussion and potentially larger studies. Although there is not a standard treatment for these bony defects, there are, however, a number of described interventions as outlined below.

There have been extensive developments in the reconstruction of critical-size defects including: 1) distraction osteogenesis 2) structural auto/allografts, 3) titanium cages and cancellous autograft, 4) polymeric membranes, 5) free microvascular fibula transplant, and 5) the use of induced membranes – the Masquelet procedure. For the sake of brevity, the authors will focus on the Masquelet procedure, polymeric membranes, and titanium cages since these procedures bear close resemblance to the described technique.

### Established techniques – Masquelet procedure

Masquelet published a series of thirty-five patients who underwent long bone reconstruction using a two-stage technique [2]. The first stage was the insertion of a polymethylmethacrylate cement spacer into the defect for two months. The spacer resulted in a pseudomembrane, which has been shown, in rabbits, to secrete vascular endothelial growth factor (VEGF), transforming growth factor-B (TGF-B), and BMP-2 [3]. Maximum BMP-2 production in the animals was demonstrated at 4 weeks post-implantation. The second stage entailed removal of the cement spacer and placement of autologous cancellous bone graft into the membrane. The lengths of bony defects reconstructed varied from 4 to 25 cm. Further studies demonstrate enhanced bony regeneration by a pseudomembrane regardless of autologous cancellous bone, suggesting that compartmentalization of the defect with a membrane alone may play a role in the regeneration process [4]. In 2002, Pelissier et al. described a forefoot reconstruction case using induced membranes and cancellous autograft with successful bone healing at 9 months [5]. More recently, Huffman et al. described a similar case using RIA bone graft and the bi-Masquelet technique [6]. Stafford also described utility of RIA bone graft and the bi-Masquelet technique for segmental bone defect nonunions [7].

### Established techniques – Polymeric membranes

In addition to the Masquelet procedure, surgeons have also utilized bioresorbable polymeric membranes. These membranes have been extensively studied in animals and used by craniomaxillofacial surgeons for bony defects since the 1970s and 1980s. Polyglycolide (PGA) was the first biodegradable polymer introduced to the medical device community. The most commonly used polymers used in orthopaedic surgery are polyglycolide (PGA), poly-L-lactide (PLLA), and the co-polymer poly (lactide-co-glycolide) (PLGA). Poly-L-lactide is degraded into carbon dioxide and water over 4 years and sometimes longer; [8] thus, to reduce the resorption rate, various co-polymers have been developed such as ε-caprolactone and glycolide. For example, PLGA membranes degrade into lactic and glycolic acid over 12 months versus 4+ years for PLA. These polymeric membranes can be designed to be heat-moldable and biodegradable with pores of varying size to allow for vascular invasion. They can be used in different configurations; i.e., molded into a tube or two membranes molded as a “tube-in-tube” neocortex construction [9]. Polymeric membranes have been shown, in animals, to encourage bony formation even without placement of autograft [10]. This bone formation has been shown due to exclusion of non-osseus tissues, maintenance of an osteogenic medullary canal, and the presence of a scaffold for periosteal regeneration and revascularization [11]. Polymeric membranes, like the authors’ technique, can be done simultaneously performed with bone grafting and internal fixation. There have been reports, however, of inflammatory reactions to biodegradable polymers that may have hindered their universal adoption. Acute *in vivo* effects of polymers include protein adsorption, mast cell histamine release, monocyte recruitment, and foreign body giant cell formation [12]. Long-term inflammatory effects of these biodegradable implants commence upon degradation of the polymer. When the host’s clearance mechanisms are overwhelmed, the chronic inflammation that ensues can potentially lead to osteolysis and fibrotic encapsulation of the implant. However, fast-resorbing PGA implants have a much higher incidence of adverse tissue reactions compared to PLLA implants.

The potential complications of polymeric degradation due to excessive inflammation have been confirmed by molecular biological studies. During normal fracture healing, research has demonstrated a biphasic inflammatory response mediated by tumor necrosis factor-α (TNF-α), interleukin-1 (IL-1), and interleukin-6 (IL-6) during the first 24 hours after initial fracture and again during the transition from chondrogenesis to osteogenesis (TNF-α and IL-1) [13,14]. In murine models, whereas low-dose TNF-α accelerated fracture healing, higher doses were inhibitory. The link between chronic inflammation with elevated levels of TNF-α and fracture healing inhibition is unclear; however, the above findings may potentially explain the more significant foreign body reaction (FBR) to PGA implants compared to PLLA implants. The PGA degradation products may incite higher levels of TNF-α.

### Established techniques – titanium cages

Titanium mesh cages, while not explored in depth in this case report, have been used successfully in conjunction with allograft to reconstruct varying lengths of long-bone defects. The first benefit of these cages is that they preclude the need for a second procedure. Secondly, they provide relatively bioinert structural support with an elastic modulus closer to bone (~105 GPa titanium alloy and ~10-25 GPa for bone); hence they offer the possibility of early load-bearing of these critical-sized defects to encourage bone formation. Lastly, because they are fenestrated, they also provide a barrier from graft extravasation while allowing for potential vascular invasion.

### Role of a membrane

The role of a membrane, vascularity and subsequent bone formation in these critical-sized defects warrants discussion. In the Masquelet procedure, upon harvest of the cement spacer, there is a pseudomembrane after 6–8 weeks. The pseudomembrane is then filled with graft and bone forms despite the fact that the membrane has no circumferential fenestrations or pores – just the opening incision for graft placement. The only circumferential vascular sources are the periosteum and the intramedullary canal. This confirms the findings of Perren that the initial phases of bone healing commence from the periosteum, proceed centrifugally with callus formation, and perhaps simultaneously, centripetally towards the medullary cavity [15]. Biomechanically, this method of endochondral bone formation is advantageous since the outer diameter of the callus increases the moment of inertia and initial stiffness of the fracture.

More recent studies have confirmed that, during the early weeks of fracture healing, the cortical bone of the intact fracture ends (near the cortical-callus boundary) undergoes resorption with a subsequent increase in cortical porosity and a decrease in elastic coefficient [16]. The authors hypothesized that the intact bone edges are demineralizing in order to: 1) aid in mineralization of the adjacent callus via calcium and phosphate sourcing, and 2) “match” the stiffness and elastic modulus of the adjacent callus to prevent local stress shielding and promote homogenous mineralization of the callus and the cortical-callus boundary.

A membrane, whether biologic or non-biologic, may contribute to this process by serving as a scaffold and/or guide for the edges of the *intact* periosteum; this intact periosteum, which is a source of osteochondral progenitor cells, can “creep” along the membrane scaffold – so called guided bone regeneration. In the case of the Masquelet procedure, the pseudomembrane may form either *around* the cement spacer as separate granulation tissue membrane or *from* existing periosteum – or a combination of both. As a membrane entity from existing periosteum, the pseudomembrane then potentially serves as a scaffold, or bed, for further periosteal and angiogenic regeneration similar to the polymeric membranes. Periosteal regeneration occurs since stem/progenitor cells have been demonstrated in the cambium layer of the adult human donor periosteum [17]. Regardless, as a new periosteum develops, it then releases growth factors as demonstrated by Pelissier et al. [3] Animal studies suggest that the periosteum may mediate the primary steps of chondrogenesis and endochondral ossification in the fracture hematoma [18]. Since the intramedullary cavity is also a vascular channel, endosteal flow may also increase after a fracture. This potential increase in intramedullary vascularity may contribute as a “supplier” of inflammatory mediators and stem/progenitor cells; however, this concept is hypothetical and could not be elucidated in the literature. Regardless, the necessity for fenestrations or pores to allow for vascular invasion - whether in cages, polymeric membranes, or the authors’ index procedures - is questioned. If they do serve a purpose, it may be to allow for possible recruitment of chondrogenic and/or osteogenic sources such as muscle-derived stromal cells (MDSCs). A recent mouse study tracking myogenic cells of the MyoD lineage suggested that these cells might infiltrate the fracture site and augment healing in open fractures with periosteal stripping [19]. Furthermore, TNF-α has been shown to recruit and induce differentiation of these muscle-derived stromal cells. The same study demonstrated that MDSCs possess more osteogenic potential than adipose and skin-derived stromal cells [20]. These studies confirm the importance of not only inflammatory mediators but also soft-tissue muscle coverage (rotational flaps, free flaps, etc.) on bony fractures at the molecular biological level. Furthermore, while not in the scope of this article, investigators have demonstrated the presence of circulating skeletal stem cells (CSSCs) present in the blood of humans and other mammals; some propose that after skeletal trauma, CSSCs may be recruited to the site of injury [21,22].

Thus, as in many complex physiologic processes, fracture healing is multi-factorial; regardless, human physiology seems to allow for redundancy in its compensatory regenerative mechanisms (periosteum, surrounding muscle, systemic/local vascularity and/or recruitment, etc).

### Advantages

The main potential advantages of the author’s technique include the following: 1) the need for only one operative session, 2) the use of a non-immunogenic graft source, and 3) the “moldability” of a Masquelet pseudomembrane while still offering the barrication/guide of other methods. The fascial graft obviates the need for a second procedure, as the patient’s own corpus is a “one-stop shop” for the majority of the reconstruction. Patients who cannot follow-up either due to non-compliance or other circumstances may be suitable candidates for such a one-stage reconstruction. In both described patients, the fascial harvest was quick to perform, and neither patient had any post-operative morbidity or functional limitation from either the ITB or RIA bone graft harvest. Since the bony defect is treated acutely, there is no repeat insult to the soft tissue or bone due to a second procedure. The rationale for the various components of the reconstruction are as follows: the fascial membrane prevents tissue intravasation and serves as a bed/guide for periosteal regeneration; RIA bone graft serves as an osteogenic source of bone marrow stromal cells, and cancellous allograft serves as an osteoconductive scaffold. In the metatarsal reconstruction case, BMP-2 served as an osteoinductive agent. A minimal amount of cancellous allograft was used as “framework” for bone regeneration; thus, it is unclear what ratio of cancellous autograft to allograft is optimal. Although marrow stromal cells (i.e., bone marrow-derived mesenchymal stem cells) have been demonstrated in numerous other tissues, the role of the fascial membrane as a potential source of stem/progenitor cells is unknown. More importantly, as mentioned before, the true value of the membrane, whether polymeric, titanium, or biologic, seems to be its role as a barricade to tissue intravasation and as a bed for periosteal regeneration. The value of the periosteal cambium layer has been clearly demonstrated and membranes seem to improve bone regeneration. For example, Reyenders et al. have demonstrated that non-vascularized periosteal autografts enhance fracture healing of bone defects in a rabbit model, especially when the graft is in contact with intact periosteum [23].

Finally, since the fascial membrane is from the patient’s own body, it is non-antigenic and thus, non-inflammatory. The flexibility and moldability of the construct is also important since it allows the surgeon to regulate the shape and length of a defect – rather than be constrained by a fixed-entity graft. Muscle-deforming forces may cause shortening, rotation, and/or angulation; thus, the surgeon can mold the graft, interpose it, and fix it in place with implants. Fine-tuning of the length, alignment, and/or rotation is afforded with a flexible graft.

As mentioned previously, polymeric membranes have both acute and long-term inflammatory effects, which have potentially hindered their widespread use. Based on the one-year post-operative CT performed on the second case, it appears that the fascial graft is still intact and not degraded.

### Disadvantages and limitations of the study

A potential disadvantage of the authors’ technique is that while the induced membranes have been shown to secrete VEGF, TGF-B, and BMP-2 in rabbits, it is unclear whether the fascial autograft does the same *in vivo*. The purpose of the graft is similar to the induced membrane – to prevent soft tissue invasion and resorption of the graft while providing a bed for periosteal regeneration. Thus, the authors would hypothesize that any potential membrane that forms on the fascial graft would potentially secrete factors. Another disadvantage of the authors’ technique is it’s potential limitation of size. The ITB, while easy to access, has limited length and width, and thus, defects of greater size may not be amenable to this procedure – although this has not been explored quantitatively. To overcome this, the graft may be meshed using a larger ratio – thus, decreasing the need for a large harvest. Another option is to utilize synthetic collagen grafts – which are commercially manufactured by numerous companies and can be custom tailored to the defect. Other authors have demonstrated union of critical-sized defects with no need for a membrane, as long as the soft tissue envelope is intact. Thus, it is unclear to what extent such membranes either “guide” bone regeneration or promote faster regeneration.

Risks of RIA bone grafting and fascial graft harvest will now be discussed. RIA bone grafting has a known described risk of subtrochanteric femur fracture [24,25]. The smallest size reamer is 12 mm, and, as the manufacturer recommends, the surgeon should pre-operatively measure the diameter of the endosteal canal to have an estimate of the size. Some patients may have a smaller intramedullary canal, and forced reaming may predispose these patients to fracture. Per verbal communication (DepuySynthes), next-generation reamer-irrigator-aspirator’s may have lower diameter reaming heads.

Another potential limitation of the technique is possible morbidity associated with the fascial donor site. Although in these two patients there was no morbidity with the fascial graft harvest, studies with larger sample sizes are necessary to establish potential complications. Autologous tissue, if associated with low morbidity, is potentially more beneficial than allogeneic, immunogenic tissue; however, as mentioned before, commercial entities may be sought after for a flexible, collagenous membrane to traverse these defects. If these membranes are effective with no immunogenic reaction, they may preclude the potential morbidity associated with a fascial harvest.

In the foot trauma case, BMP-2 was utilized whereas it was not utilized in the second case. There was no specific rationale for its use or dosage in the first case, apart from being an osteoinductive agent, since the utility of it was off-label. More recently, BMP-2 has demonstrated to be effective at lower *in vivo* doses (verbal communication, Medtronic). The potential off-label risks of BMP-2 have been documented in the literature and include: ectopic bone formation [26], swelling/hematoma [27], neoplasia [28], and wound problems; [29] this should be taken into consideration when using these therapies off-label. As basic science researchers, translational scientists and surgeons further understand the temporospatial orchestration of growth factor/cytokines during fracture healing, more precise delivery, timing and dosing of these factors could be delivered to these defects.

A last factor to consider is the appropriate apportionment of osteogenic and osteoconductive sources. We utilized a low volume of cancellous allograft in both cases, since the authors could not determine the ideal combination of osteoconductive (cancellous graft) and osteogenic/osteoconductive (RIA) for optimal bone regeneration. Since the RIA bone graft contained both osteoconductive, osteogenic, and osteoinductive elements, we utilized more of this component than the cancellous allograft. The allograft theoretically served as a structural scaffold for osteoblasts and their precursors.

### Future directions

The authors’ method offers a moldable, autologous, and non-immunogenic method to aid in the reconstruction of small-bone (radius/ulna/metatarsals), and potentially large-bone (femur/tibia/humerus) critical-sized bony defects. As our understanding of fracture healing progresses, orthopaedic surgeons, stem cell biologists, and mechanobiologists may seek additional solutions to recreate the anatomy – especially when the defect involves the articular surface. For example, in the forefoot reconstruction, a potential alternative solution would have been to harvest articular cartilage, with its accompanying tidemark, from the distal femur. This cartilage/tidemark graft is then placed, or “contoured” in the proximal portion of the metatarsal graft (in addition to the RIA) to serve as an articulation with the midfoot. The flexibility of the fascial construct is important since it affords the surgeon a moldable, biologic solution offering increased surface area contact with the native anatomy; i.e., periosteum.

However, newer digital modeling and fabrication technologies incorporating computer numeric control (CNC) machines (biologic 3D printer) may supplant existing structural grafts (either rigid or flexible) in an effort to more accurately recreate native human anatomy [30,31]. For example, a new custom-fabricated metatarsal, made from non-immunogenic osteoconductive material such as hydroxyapatite and/or collagen type I, can be designed based on the patient’s contralateral imaging data (i.e., a 3D CT of the normal extremity/bone). Custom-made biodegradable stereolithographic resins can be “loaded” with a source of stem/progenitor cells as a potential future option for these defects. Synthetic collagen and/or hydroxyapatite scaffolds can be further treated with agents (i.e., RGD peptide (Arg-Gly-Asp) [32], cell-binding domain of Collagen type I P-15, bone sialoprotein [33], etc.) that promote osteoblast adhesion and/or differentiation. This newly engineered graft can then be pre-loaded with autologous bone graft and then placed in the defect with fixation of choice. Whether such newer technologies would be cost-effective is unknown. And, in an era of significant expense for newer technologies, cost-efficacy with a large series of cases would be warranted.

**Figure 14:**
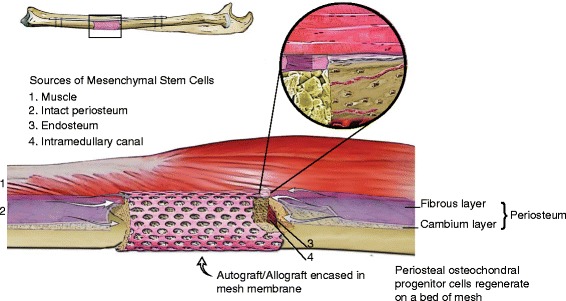
**Illustration demonstrating placement of fascial graft construct with potential sources of stem/progenitor cells.** Illustration courtesy of Bernie Kida, M.F.A., Atlanta, Georgia.

Despite our increased understanding of fracture healing with newer tissue engineering techniques, many unknowns still exist. For example, in a critical-sized long-bone defect, how do the fracture fragments “communicate” with each other – with or without auto/allograft? The intercalary graft in this article “communicates” with the proximal and distal intact radial shaft segments both intramedullary, cortically, and periosteally. But how does the physiology “know” where to create a cartilaginous intermediate with subsequent formation of osteoid and mineralization? Are there chemokine gradients or trophic mediators that incite periosteal regeneration with subsequent stem cell differentiation and bridging callus? How much “micromotion” (i.e., micrometers, millimeters) stimulates enchondral bone formation and does it depend on the fracture pattern and/or bone involved? For example, long oblique fractures may tolerate more motion or strain whereas transverse fractures are less tolerant. Comminuted fractures and the defects discussed in this article seem to benefit from motion but the reasons are unclear, whether it is from fluid-flow based mechanotransduction (prostaglandin E_2_/I_2_, nitrous oxide), streaming potentials, etc. Regardless, based on the ability of bone to regenerate across large bony defects, it is clear that there are additional factors involved that we still do not fully understand. And many of these factors need further research to facilitate effective intervention (Figure 14):

1. Temporospatial cascade of inflammatory (TNF-α, IL-1, IL-6)/differentiation (BMP-2, BMP-6, BMP-7, etc.) mediators. As mentioned, this area of research has provided a deep understanding into the orchestration of fracture healing, but is clearly not the only factor – especially in large bony defects.
2. Periosteal and endosteal scaffolds comprised of Collagen type I and/or hydroxyapatite. These scaffolds serve not only to guide bone regeneration to a specific anatomic shape; they also serve as osteoconductive materials due to presence of RGD domains and other cell adhesion molecules (CAM) to enhance osteoblast adhesion/differentiation. As mentioned before, scaffolds that are pre-treated with adhesion and/or differentiation substances – a so-called “supercharged” or “sticky” scaffold – may also expedite the process. It is unclear, though, whether a periosteal and/or endosteal bridging scaffold augments or expedites the healing process. In other words, would these index fractures have healed without the fascial graft, i.e. with RIA bone graft alone, but perhaps taken longer or resulted in a nonunion?
3. Stem/progenitor cells serve as the osteogenic source of mineralization. Their local presence and/or delivery/recruitment to the defect are critical to this process. Although still poorly understood, CSSCs may also play a role in this process. Vascularity is obviously a critical factor in local delivery of mediators and cells; and although no mentioned of oxygen tension has been made, this factor is also a significant component to the fracture healing process.
4. Piezoelectric phenomena were always thought to guide fracture healing. Over the past few decades, however, research has indicated that fracture healing may be mediated by both piezoelectric (“dry” bone phenomena based on collagen fibrils) and streaming potential (“wet” bone) phenomena. The precise transformative mechanism of bioelectric phenomena to molecular biological events is largely unknown. A full discussion of these phenomena are outside the scope of this article, but there may be a more precise and effective manner of harnessing these processes.

The precise contributions and “bottleneck” steps in endochondral bone formation have yet to be elucidated: the role of periosteal regeneration with/without a scaffold, the temporospatial cascade of inflammatory and differentiation mediators, vascular sourcing and recruitment of MSCs with pericytic/angiogenic invasion, streaming potentials/fluid flow, oxygen tension, pH, etc. In order to dramatically affect and hasten fracture healing beyond standard human physiology, a multi-factorial approach, as opposed to a “one-pill wonder,” will likely be necessary – one in which the limiting steps to the process are understood and augmented accordingly. These limiting steps may be custom-tailored to the patient’s physiology – either inherent genetic makeup and/or acquired disease (i.e. vasculopathic, neuropathic, osteopathic patients due to genetic makeup or disease processes).

Current and future research is directed at a deeper understanding of the above concepts with an emphasis on newer means not only to achieve bony union but moreso to hasten the process:

“I not only think we will tamper with Mother Nature. I think Mother wants us to.”– Willard Gaylin

## Conclusion

This paper demonstrates a one-stage procedure using cancellous RIA autograft encased in a fenestrated fascial autograft as a biologic membrane to aid in reconstruction of critical-sized bony defects. The potential benefit of a fascial autograft membrane for critical-sized bony defects versus other current technologies was discussed. The authors’ technique potentially warrants future research as another option in the surgeon’s armamentarium in managing these difficult injuries.

## Consent

Written informed consent was obtained from the patient for publication of this Case report and any accompanying images. A copy of the written consent is available for review by the Editor-in-Chief of this journal.
